# Collective minds: social network topology shapes collective cognition

**DOI:** 10.1098/rstb.2020.0315

**Published:** 2022-01-31

**Authors:** Ida Momennejad

**Affiliations:** Microsoft Research NYC, New York, NY, USA

**Keywords:** network topology, collective cognition, human memory, collective memory, cognitive neuroscience, social networks

## Abstract

Human cognition is not solitary, it is shaped by collective learning and memory. Unlike swarms or herds, human social networks have diverse topologies, serving diverse modes of collective cognition and behaviour. Here, we review research that combines network structure with psychological and neural experiments and modelling to understand how the topology of social networks shapes collective cognition. First, we review graph-theoretical approaches to behavioural experiments on collective memory, belief propagation and problem solving. These results show that different topologies of communication networks synchronize or integrate knowledge differently, serving diverse collective goals. Second, we discuss neuroimaging studies showing that human brains encode the topology of one's larger social network and show similar neural patterns to neural patterns of our friends and community ties (e.g. when watching movies). Third, we discuss cognitive similarities between learning social and non-social topologies, e.g. in spatial and associative learning, as well as common brain regions involved in processing social and non-social topologies. Finally, we discuss recent machine learning approaches to collective communication and cooperation in multi-agent artificial networks. Combining network science with cognitive, neural and computational approaches empowers investigating how social structures shape collective cognition, which can in turn help design goal-directed social network topologies.

This article is part of a discussion meeting issue ‘The emergence of collective knowledge and cumulative culture in animals, humans and machines’.

## 1. Introduction

Human cognition is not solitary. From tool use, language and mathematics to beliefs about the world and morality, human cognition is shaped by learning and memory in social networks. Unlike swarms or herds, human social networks have diverse structures comprising strong, weak, clustered and sometimes hierarchical ties that serve different roles. Networks of humans pass and share information in order to synchronize their collective memories, knowledge and beliefs, or to discover and integrate diverse information and knowledge. This review focuses on empirical and computational investigations of how the structures of communication networks (i.e. social network topology) shape collective cognition. Specifically, we ask how social network topology interacts with psychological, neural and computational principles of learning and memory to synchronize collective memory and knowledge.

While a number of prominent papers and books over the past decades have addressed the role of cooperation, and social networks on collective outcomes, a review with the particular synthesis of social network topology with psychological and neural computation seems lacking. Thus, this review narrows the focus to notable research on the interaction of network structures (macro level) with psychology and cognition (micro level) in behavioural studies of collective memory [1,2], collective beliefs and behaviour [3–5], collective discovery and cultural accumulation [6], the neuroscience of social and non-social navigation of large networks [7,8], and the emergence of communication in machine intelligence and multi-agent systems [9–11].

Before returning to the focus of the review, it is noteworthy that over the past decades diverse disciplines have investigated different faces of collective cognition. Philosophers such as Bratman [12–14], Davidson [15], Tuomela [16] and Gilbert [17,18] have pioneered theories of shared agency, collective beliefs and shared intentionality. Highlighting the importance of collective beliefs and intentions on actions, this philosophical work bridges notions of sociality to morality and ethics. Anthropologists have investigated the evolution of cooperation and cumulation of culture [6,19], as have developmental and comparative psychologists focusing on primate and infant theory of mind, shared intentionality and cooperation [20–24]. Also noteworthy is related research on shared intentionality, reason-giving and the evolution of human culture; see O'Madagain & Tomasello in the present special issue [25]. Other psychologists have combined modelling and experiments to investigate cooperation [26], the emergence of groups based on reciprocity and transitivity [27], and the conditions under which a pair of humans outperform the best of the two in perceptual decisions [28]. A comprehensive review of this literature is outside the scope of the present manuscript and requires a larger review integrating and bridging the present perspective with traditions in philosophy, anthropology and decision science.

Studying the topology of human communication networks empowers us to understand, explain, model and predict the emergence and dynamics of collective knowledge in large networks. Decades of seminal research by renowned mathematicians, physicists, neuroscientists, computer scientists, sociologists and economists have established the science of complex networks that are brilliantly reviewed in earlier publications [29–33].

This manuscript specifically focuses on the combination of network topology research with the methods of computational and cognitive sciences. A graph-theoretic understanding enables us to study how communication networks interact with psychological and neural computation to shape collective cognition. Moreover, it enables us to make goal-directed predictions, and design interventions to achieve desired collective cognitive outcomes. Such desired collective outcomes could span from predicting and combating misinformed beliefs about a global pandemic to facilitating optimal structure of classrooms for learning, synchronizing memories prior to elections, optimally connecting scientific task forces working on rapid vaccine discovery, studies of human collective cognition empowering researchers and designing effective multi-agent machine intelligence.

This paper reviews recent directions of studying collective human cognition within the scope established above and concludes with a brief discussion of current and future directions in multi-agent machine intelligence. We review how network topology aligns collective memories (§2), collective beliefs and behaviour (§3), cultural accumulation and collective intelligence (§4). We then discuss how the brain's neural responses capture the topology of one's social network (§5) and then discuss common neural findings in cognition of social and non-social topologies (§6). We close with applications in multi-agent machine learning (§7) and a summary of the topology of social networks in humans and machines (§8).

## 2. Network topology aligns collective memory

**Figure 1.**
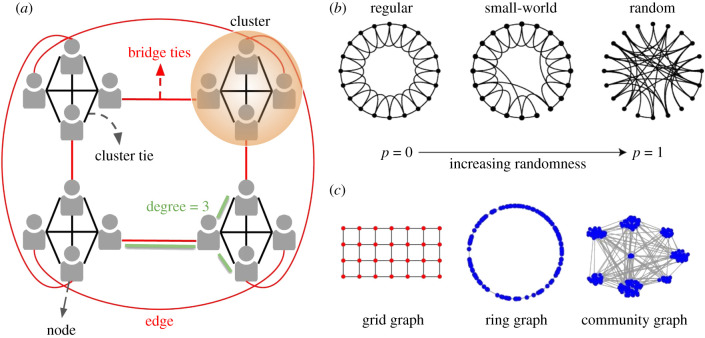
A primer on network topology. Social and non-social networks can be analysed in terms of graphs. A schematic of the network topology from a study on collective memory [2] is displayed (*a*). Nodes, denoting people in a community, are depicted with human graphics. Edges, denoting direct communication between two nodes or persons, are illustrated as lines. Clusters, bridge ties among nodes that do not have common connections in common, as well as cluster ties among nodes that have many common ties are marked. The degree of a node, i.e. the number of its ties, is marked in green. Standard parameters that vary the structure of graphs include randomness (*b*), clustering (*a*,*c*), network diameter (maximum path length) and average path length. A number of well-known graph topologies are depicted: a graph with a grid structure, a random ring graph and a network with a clustered community structure (*c*).

A key question in understanding collective cognition is how the structures of communication networks (figure 1) align collectively shared memories and beliefs. From friendship circles to large communities, shared memories often shape group identity, which in turn facilitates collective action. There are different definitions of collective memory in the social and psychological literature. In this paper, collective memory refers to the *convergence of memories among the members of a social network or community* [1,2]. However, there are other notions of collective memory in the social sciences, such as public symbols maintained by societies [34,35], e.g. war memorial monuments, among others.

In a number of studies, Coman, Momennejad and colleagues investigated collective memory using graph theory, behavioural experiments, complex and temporal network analysis, and agent-based simulations [1,2,36]. To do so, they designed laboratory-based controlled experiments on collective memory, during which participants were assigned to pre-arranged communication networks in a virtual setting. They devised novel behavioural analyses, inspired by representational similarity analysis in cognitive neuroscience, to measure mnemonic convergence in social networks when individuals were not aware of the broader topology [1,2].

**Figure 2.**
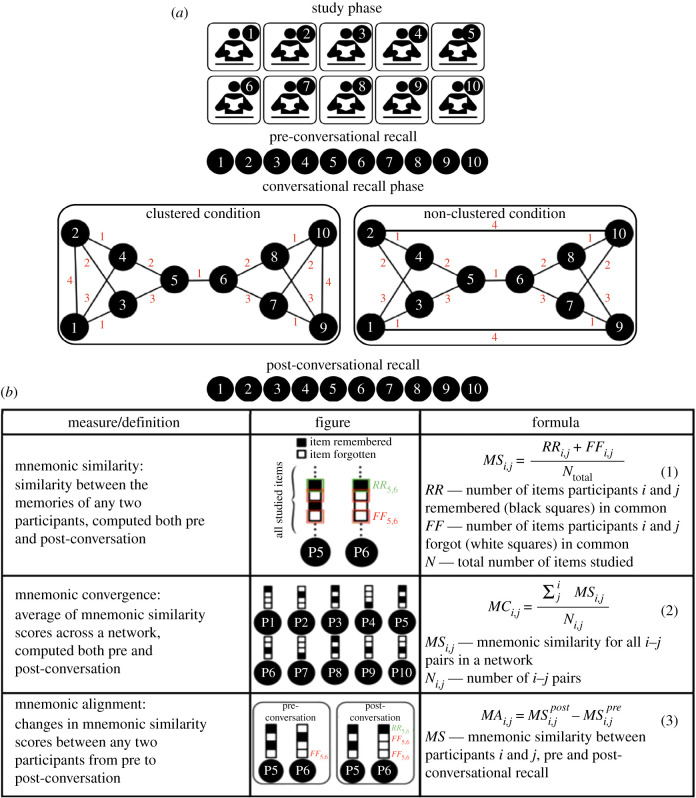
Studying the effect of network topology on collective memory. (*a*) Experimental design for measuring collective memory in a laboratory-formed communication network [1,34]. Unbeknownst to the participants, we assigned a 10-person topology to each experimental session with three phases: a study phase with an individual recall test, a conversation phase and a post-conversational individual recall test. The numbers on graph edges reflect the order in which a conversation between those two persons occurred. (*b*) Equations for computing how similar the memories became as a function of conversations. Members of the network are identified with a number, e.g. P6: person 6. (Online version in colour.)

Every experimental session consisted of a number of individuals (e.g. 10 or 16), each facing a computer screen and later interacting with a pre-assigned number of other participants virtually through a text chat window. All experiments followed a three-phase design (figure 2): in phase 1, participants studied the same material individually and took a memory recall test (pre-conversational recall), in phase 2, each participant had a series of dyadic conversations through a pop-up chat window during which they could discuss what they remembered from the material they had just studied. Participants did not see who they were talking to, nor were they aware of the larger network structure. In phase 3, each participant took a recall test once again individually (post-conversational recall). Having pre- and post-conversational behavioural measures of memory recall allowed us to measure mnemonic convergence owing to conversations, in two different topology conditions: clustered and non-clustered topologies (figure 2). A series of representational similarity analyses (figure 2) was designed and conducted, inspired by similar techniques in neuroimaging, to compute and compare individual and collective memory in relation to network topology.

The authors first coded the behavioural memory recall responses from phase 1 and phase 3 as vectors of recall items. For instance, one study had 16 recall questions [1], which resulted in two 16-item vectors for pre- and post-conversational recall. Each item coded 1 if the participant recalled the item correctly, and 0 otherwise. Then *mnemonic similarity* (figure 2) was computed for every pair of participants, this was computed as the correlation or dot product among their recall vectors during pre-conversational and post-conversational phases. *Mnemonic similarity* measures how similarly a pair of participants recalled and forgot items. Then all pairwise similarities were averaged to compute the *mnemonic convergence* of the network, or how similarly the entire network recalled and forgot the items (figure 2). Subtracting mnemonic similarity before and after conversations, *mnemonic alignment was computed*, or the extent to which two participants' memories (both recall and forgetting) became more similar after conversations. Mnemonic alignments of all pairs were averaged to measure the collective memory of the network.

Using the experimental paradigm described above (figure 2), the authors studied the effect of the structure or topology of a network on how convergent participants’ memories became after conversations, a measure of collective memory convergence [1]. Data were collected from 10-person networks, in which each member had three conversations either ordered according to a clustered topology or a non-clustered network topology. The authors measured eight 10-person networks in the clustered and eight 10-person networks with a non-clustered topology condition (figure 2).

**Figure 3.**
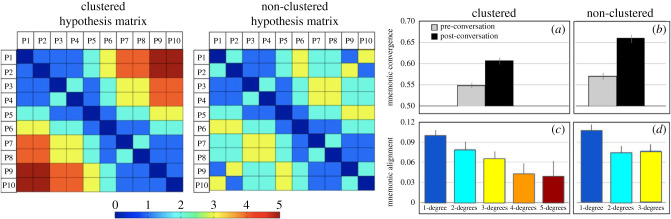
The effect of network topology on collective memory alignment. Mnemonic similarity hypotheses matrices corresponding to the clustered topology condition and the non-clustered condition in the experiment described in figure 2 are displayed. Members of the network are identified with a number, e.g. P6. The similarity scores range from 0 (distance to self; dark blue) to 5 (greatest degree of separation; dark red). Behavioural results show that on average, memories aligned more after the conversations in the non-clustered network (*b*) compared to the clustered network (*a*). This finding can be explained by the hypothesis matrices, suggesting that alignment depends on the degree of separation. Because the clustered network topology has larger degrees of separation (i.e. longer geodesic distance or network diameter), it leads to lower convergence. The extent of this alignment depends on how many degrees of separation they are from one another in the social network (*c*,*d*). (Online version in colour.)

The hypothesis was that pairwise participants' memories would align according to their degree of separation, with the most alignment in those with a direct conversation and the least alignments with those with the further geodesic distance (figure 3). This hypothesis was confirmed in the behavioural measurements of alignment (figure 3). The pairwise results had a consequence for the collective memory of the larger network as well. Networks with clustered topologies had a higher *network diameter*, or a longer path between the most distant members of the network, than the non-clustered network. The results show that the collective memory of networks with a smaller diameter (non-clustered topology) converged more than the networks with a clustered graph structure or topology.

In another experiment [2], Momennejad and colleagues showed that given a fixed communication network topology, the temporal order in which communications unfold over time determined the extent of memory alignment. Namely, consistent with Granovetter's proposal about the strength of weak ties [37], they found that collective memories converged more if individuals with weak or *bridge* ties exchanged information first. These were individuals who had a direct tie but did not have any ties to other individuals in common (an analogy to intuit their network status would be if they were friends who talked, but their friends weren't friends and didn't talk to one another).

The studies above show that the topological structure and temporal order of communication networks determine the mnemonic alignment among individuals in a network, even if they did not directly interact. In a recent computational study [36], we combined multi-agent or agent-based simulations with models of retrieval-induced forgetting from the psychology of memory [38,39] and were able to simulate these behavioural findings in collective memory. Together, these behavioural and computational studies offer a quantitative approach to measuring the emergence of collective level or meso-scale phenomena from network topology and principles of memory and forgetting.

These recent advances carve the way for theoretical and experimental approaches to studying the ties that bind the micro, meso and macro scales of human cognition and behaviour. Graph-theoretic approaches to collective experiments, and simulation of institutions [40] with parameters based on experiments, can help understand, predict and compare the behaviour of different topologies of human networks.

## 3. Network topology shapes beliefs and norms

Collective memories often form the foundations of shared systems of beliefs, and beliefs shape how we remember [41]. Religion, political choices and health decisions are among key examples of how shared beliefs, and their changing dynamics, guide individual and collective decision making. Computational simulations have established how beliefs spread in social networks [42]. Recent empirical evidence suggests that collective beliefs can be synchronized according to a social network's topology [43].

Vlasceanu and Coman conducted a belief synchronization version of the study described in figure 2 [1,5]. They showed that, similar to the collective memory counterpart study [1], the topology of social networks impacts the alignment of beliefs. This belief alignment occurred even among members who never directly communicated and the extent of change in belief similarity between two individuals corresponded to their degree of separation in the network.

The authors also studied how belief endorsement by a public speaker affects belief alignment in 12-person laboratory-created networks [43]. During the experiment, individuals first evaluated how believable a set of belief statements were and later either completed a distractor task (control condition) or listened to a public speaker who endorsed some of the beliefs (treatment condition). The authors showed that the public speaker altered the mnemonic accessibility of some beliefs, which in turn impacted and was amplified by the networks' conversations, leading to subsequent belief synchronization. Future studies are required to study how an individual's network affinity with the public speaker shapes the direction of their influence.

The present manuscript focuses on the role of social network topology in collective cognition rather than behaviour broadly defined. However, beliefs about health, education and politics govern large-scale patterns of collective human behaviour. Thus, it is reasonable to hypothesize that the topology of communication networks shape behaviour. Centola has provided evidence for this hypothesis in a series of seminal studies, establishing a link between network topology and collective behaviour [3,4]. They investigated the mechanisms by which health behaviour would spread in social networks and identified which topologies were more conducive to the adoption of health behaviour. They found that individuals were more likely to adopt health behaviour if they received reinforcement about that behaviour from many close ties in the broader network. Therefore, health adoption spread more effectively in clustered lattice network topologies, compared to random networks [3]. In a second study, Centola investigated how the composition of a community affects the adoption of health behaviours. They discovered that *homophily* between two individuals, i.e. the extent to which their social contacts are similar to one another, increases their dyadic-level influence on each other's behaviour. However, the broader consequence of this pattern is that in large clustered networks less healthy individuals are more likely to have close ties and influence one another, reducing their probability of interactions with, and being influenced by, healthier individuals [4].

While the present manuscript is a review of the role of network topology in collective cognition with a focus on cognitive neuroscience and machine learning, a series of recent papers have addressed how topology affects health behaviour, resource sharing and inequality, and wisdom of the crowds (i.e. the observation that the average response of a group better approximates ground truth than individual responses) [34–39]. These are related, fascinating and important topics but outside the scope of micro–macro interactions in collective cognition here, yet a number of papers on network approaches to changing social norms are particularly noteworthy. Banerjee and colleagues studied the diffusion of microfinance in villages, showing that if information is first given to central individuals (measured by their eigenvector centrality in the social network), adoption of the information diffuses more effectively in the community [44]. In seminal research, Paluck and colleagues conducted anti-conflict interventions across over 24 000 students and showed that individuals pay more attention to ‘social referent’ network roles (influential individuals) in their community and interventions are more effective when targeted on referents, reducing conflict by 30% in 1 year [45]. This was followed by further research into engineering social change: temporal- and network-based improvements of norm change, nudging and attitudes to authority and justice in factory workers [46–49].

Taken together, these studies clarify the role of network topology in the mechanisms by which beliefs, norms and behaviour propagate in networks, and how centralized interventions—such as collective exposure to public endorsement and endorsement by influential members—can change collective beliefs and norms. These findings, together with findings on collective memory and belief formation, offer the important potential for designing interventions to combat misinformation (e.g. about health behaviour) or harmful polarization in larger networks. Mapping network topology of misinformation networks makes it possible to devise a number of intervention tools that can synchronize memories and beliefs in larger communities at times of crisis. These tools could span from affecting the topology of communication networks, when possible, to targeting bridge ties and isolated clusters (information bubbles) for intervention and centralized public speeches.

## 4. Network topology shapes collective intelligence

Consider the Brooklyn bridge, your smart phone, or the international space station. Achieving any of these complex technologies required generations of cumulative inventions, leading to levels of problem-solving ability that go far beyond what is possible at the individual intelligence level. It has been suggested that the capacity and success of cumulative cultural achievements, such as complex technologies, depend on the size and connectivity of collaborative networks [6].

In an inventive study, Derex & Boyd [6] investigated how the topology and temporal order of collaboration networks lead to inventions. They studied six-person collaboration networks, attempting to discover three hierarchical levels of chemical compounds in a virtual set-up. In one condition, all six members of the network worked together simultaneously (*full connectivity*), while in the second condition, they worked in three teams of two and exchanged members twice throughout the experiment (*partial connectivity*). The results suggest that participants in the partial connectivity condition achieved further levels of the hierarchical invention compared to the full connectivity condition.

Thus, while full connectivity may serve the convergence of memories and beliefs, as discussed earlier, partial connectivity may increase cultural accumulation required for complex and cumulative invention. These findings provide compelling support for the core thesis of the present paper: that different social network topologies can serve different collective outcomes. Further hypothesis-driven investigations could lead to insight for intervention design: designing goal-directed network structures or targeted interventions on specific parts of a network towards a desired outcome. Examples of desired collective outcomes span from predicting and combating misinformation about a global pandemic to designing optimal communication among scientific research networks for rapid vaccine discovery.

Wooley and colleagues define collective intelligence as the above-mentioned collective's ability to solve a diverse set of tasks and problems beyond the ability of the most intelligent members of the collective [50,51]. They have conducted a number of studies investigating how the composition and diversity of teams can enhance its measurable collective intelligence and help achieve optimal goal-directed problem solving [50,51]. They show that the social sensitivity of team members, as measured by the social intelligence test of detecting emotions from photographs of eyes [52], predicted the collective intelligence of the teams. However, this measure of social sensitivity does not measure individuals' sensitivity for encoding the broader relational topology of the social network.

The study by Derex and Boyd discussed earlier provides preliminary evidence for the hypothesis that network topology and connectivity can impact the collective intelligence of a community. Other studies have established collaborative learning in social networks [53] and investigated the role of team size and composition on complex tasks [54]. However, as we have seen, different network topologies afford optimal solutions towards different goals. One hypothesis for future studies is that *the collective intelligence of a collaboration network may depend on the network's flexibility in reorganizing its collaborative connectivity to adapt to diverse task demands*.

Another future direction is to investigate whether collective intelligence merely relies on sensitivity to social cues, or whether it is also related to the ability of community members to perceive the network status of others, and the broader topology of their social network. Such a study can benefit from the methods developed in recent neuroimaging studies (discussed in the following section) showing that the brain spontaneously encodes the social network status of one's community members.

## 5. Social network topology shapes neural responses

Previous sections reviewed behavioural evidence that human memories and beliefs become similar to one's community members, and the extent of this similarity tracks one's geodesic distance, or degree of separation, to any other community member. Given these behavioural findings, a hypothesis naturally follows: social network topology should also impact similarities in the brain signals of community members. A recent study considered whether human brains are sensitive to the network status of other individuals [7]. The authors' reason that because human social groups, unlike herds and swarms, comprises diverse bonds and structures, human brains might have evolved to endure the cognitive demands of navigating complex social networks. This means being able to track social ties and relationships that are direct (one step away), third party or more distant (multiple steps away)—extending to an understanding of the broader social network topology.

The study used graph-theoretic measures such as *eigenvector centrality* to analyse an academic cohort of 275 students and scanned 21 members in a functional magnetic resonance imaging (fMRI) study. Eigenvector centrality measures how influential a member is and how many influential members they are connected to (which is different from degree centrality, counting how many connections a given individual has, figure 1). During the study, each person viewed photos of other individuals with varying degrees of separation from themselves. This included individuals with eigenvector centrality, as a measure of *influence*, and individuals who were bridge ties between otherwise unconnected members of the cohort (figure 1), as a measure of *brokerage*. Neural pattern analysis revealed that while each individual viewed photos of their cohort, their brain activated the network position of the individual they were viewing. Notably, this neural representation of the network position of cohort members was activated spontaneously in the brain, i.e. in the absence of an explicit goal that required this knowledge. This is in line with the hypothesis that human brains might have adapted to encode the topology of social network ties beyond one's immediate bonds [55].

A series of fMRI studies have studied similarities in the brain activation of members of a community as they each watched videos inside an fMRI scanner. The researchers had mapped the graph of the social network of the individuals, measured the network status of different members in terms of different measures of centrality and analysed a relationship between similarities in brain responses videos and the social network measures of closeness. One such study [56] reported that the neural responses of individuals during audiovisual video viewing were more similar to neural responses of their close community ties. The extent of this neural similarity tracked the pair's distance in a social network: friends with smaller geodesic distance had more similar brain responses while community members with more degrees of separation showed less similar responses. Another study showed that the community members' brain activities while watching videos became more similar to members of the network with high eigenvector centrality, i.e. highly influential members, what the authors dubbed ‘neural influence’ [57].

We have so far reviewed graph-theoretic studies of the synchronizations of collective memories [1,2], behaviour and beliefs [43] in social networks; the alignment of neural similarity among members of a community [56,57]; and the neural encoding of the network position of one's community members [7]. These studies suggest a broader human capacity for learning network structures in social and non-social cognition.

## 6. Navigating social and non-social topologies: common mechanisms?

Human social groups are larger than those of our evolutionary cousins, and the social network size is proposed to correspond to the size of the brain in primates [55] and other mammals [58]. Unlike herds and swarms, human communities comprise social networks with diverse structures. A number of papers in the present special issue focus on cumulative cultural evolution and the structure of populations as the origins of moving from foraging to collective intelligence, while others shed light on the social networks of hunter–gatherers to understand cultural evolution (see [59,60]; as well as [61]). While the burgeoning research on social learning across the species points at cultural evolution and collective knowledge (see Garland *et al*. [62]; Gruber *et al*. [63]; Whiten *et al.* [64]; Wild *et al*. [65]; and Williams & Lachlan [66] in the present special issue), this manuscript particularly focuses on the role of network topology on collective cognitions in humans, leaving out the evolutionary framework. That said, it is reasonable to hypothesize that human brains have evolved to handle the cognitive demands of navigating complex social networks, and that vice versa, perhaps the demand of adapting to the growth of social networks contributed to broader practices and cultures of learning and navigating complex networks. While it remains unclear how neural and cultural capacities for graph learning in social, spatial or associative contexts have co-evolved, and which was prior, it is helpful to consider common tools and findings across studies of structure learning [8].

A number of behavioural, neuroimaging and computational studies on associative, statistical and representation learning have identified the human capacity for learning multi-step topologies and community structures of sequences [67–69]. Both connectionist and reinforcement learning (RL) computational modelling frameworks have offered accounts of how the brain may generalize associative learning of sequential structures into learning of larger structures [8]. Recent behavioural and neuroscientific research have identified the computational learning principles of social structure learning [70] and brain networks underlying such learning [68,71,72].

Let us consider a number of studies that paint a broader picture of the human capacity for learning and navigating non-social network structures. Studies have shown that statistical learning of sequences underlies how humans learn and represent graphs and networks in eight-month-old children [73], in extracting statistics of temporal events in associative learning of higher order temporal structures [74], and that similar principles can be generalized spanning to language acquisition [75,76]. Others have investigated individual differences in learning social and non-social structures [77], and how the learning of local patterns gives rise to learning of network topologies [78,79].

More recently, Schapiro and colleagues have shown that humans implicitly learn the larger structure of a network as they view a sequence of individual stimuli. Using fMRI, they showed that this graph learning is represented in the prefrontal cortex (PFC) and medial temporal lobe regions of the brain [67]. This paradigm has been adopted by other researchers to study how humans learn statistical structures with different topologies [80]. Moreover, a series of behavioural and neuroimaging studies have used the RL framework, providing evidence for learning multi-step associative relations structures (or successor representations) [68,69,71,81], which may underlie how the brain learns topological structures of social networks as well. Recent human neuroimaging supports the idea that novel inference of social hierarchies relies on neural mechanisms similar to those in navigation.

The similarity of social topological learning to navigating spatial topologies, finding shortcuts and learning non-spatial associative topologies calls for more comparative studies of common mechanisms. Two particular brain regions involved in social, spatial and other modes of topological learning are the medial PFC (mPFC) and the hippocampal-entorhinal complex [72,81–84]. Of the brain regions discussed earlier, the mPFC differs the most between human and non-human animals, with anterior parts of the mPFC in particular associated with social cognition. Anterior mPFC (Brodmann area 10) has also been implicated in representing prospective tasks while performing a different ongoing task as well as multitasking [85–87], analogical reasoning [88] and social reasoning [89–91].

Notably, these studies largely draw from expertise and diverse tools from neuroscience, mathematics, graph theory and physics [92]. Identifying common brain networks that underlie the human capacity for cognition of social and non-social topologies can offer insight into understanding collective cognition and cultural evolution. Interdisciplinary experimentation and modelling could help elucidate the dynamics of the coevolution of the human brain's capacity for learning social and non-social networks. In turn, understanding the neural computational capacities of human and non-human primate brains for learning graphs and topologies can inspire multi-agent architectures for collective machine intelligence.

## 7. Application to collective machine intelligence

A thriving direction in contemporary machine learning regards multi-agent learning and collaborative artificial intelligence (AI). Research and innovation in these directions span from AI-AI and AI-human interactions, including communication via natural language processing [93], to building AI tools for enhancing human–human interactions [94]. This journal issue includes a number of such directions, such as experiments in artificial culture in collective social robotics (see [95]), as well as research on embodied evolution of social learning in swarm robotics (see [96]) and artificial evolution of robot bodies and cultural learning (see [97]). Recent advances range from deep RL agents that play computer games such as project Malmo, Xbox games, and Minecraft [9,98] and Starcraft [11,99] to multi-agent networks that evolve communication-based social influence [10], interaction-grounded learning [100] and interactive meta-learning [101]. Agent-interaction graphs have been used to evaluate generalization in multi-agent systems [102]. Future directions of multi-agent machine learning can combine insights from brain networks and human social networks to both help understand human collective cognition, and advance collaboration and collective machine intelligence.

A forward-looking computational direction is to compare the emergence of different social structures dependent upon the neural architecture of individual agents in a multi-agent system. It is possible to envision at least two related directions. First, the emergence of *optimal network topologies tailored to fulfilling particular tasks or goals* in a multi-agent setting. The goals of such a system could vary from collaboration to competition (e.g. as in Xbox games) or assisting humans. Second, combining principles of evolving network architecture with multi-agent problem settings can offer insights into the coevolution of neural architecture (in individual agents) and the topologies of social and ecological multi-agent networks. These directions are especially timely given recent advances in graph learning [103].

Advances in these directions could offer theoretical insight into the correspondence and coevolution of neural architectures in individual brains and species with the demands of navigating large and complex social, spatial and environmental networks. Which structures of social networks emerge from multi-agent systems with different goals? Which neural network or brain architectures can afford the multi-agent behaviour observed in a given species? This direction offers exciting prospects for studying how neural networks and social networks co-evolve in biological and AI.

## 8. Conclusion: the topology of collective cognition in human and machines

Human brains and cultures are embedded in large social and ecological systems. Unlike swarms and herds, human social networks have diverse composition and topologies. Here we have reviewed research backing the hypothesis that social network topology shapes collective cognition and behaviour. We narrowed the scope predominantly to psychological and neuroscientific studies that ground this proposal in micro–macro interactions.

The integration of these studies shows that: (i) human memories, beliefs and behaviours synchronize with their social ties, and with members of the community they never directly communicate with [1–5]; (ii) human brains spontaneously process the network status of others in one's social communities, and the similarity of brain responses while watching movie clips predict friendship ties within a cohort [7,56]; (iii) the brain's ability to encode the broader network topology beyond one's immediate ties mirrors the brain's ability to learn non-social topologies and cognitive maps [104]. We reviewed evidence from the neuroscience of learning and memory pointing at potential common mechanisms for learning social, spatial and non-social topologies [8,67,77]: (iv) recent deep learning algorithms connect this literature to collective cognition in multi-agent machine learning [10]. This diverse body of research supports the hypothesis that the brain's ability to acquire and navigate topologies of complex and large neural networks might have co-evolved with the human species' growing network size and diversity of social topologies. Research on social and affective disorders could elucidate commonalities and differences in social and non-social graph learning.

The body of research reviewed here uses diverse interdisciplinary methods developed in graph theory, statistics, mathematics, physics and neuroscience for clustering and characterizing community structures in complex networks [105,106], temporal network analysis in dynamic complex systems (for instance when analysing the effect of the order of conversations on collective memory) [2] and representational similarity analysis for comparing multidimensional vectors (e.g. for analysing neural patterns [84]) adapted for comparing mnemonic convergence in behaviour, using the correlation of multi-item memory vectors [1,34].

Graph-theoretic tools for analysing the architecture of complex networks apply to brains and social networks alike. Just as neural networks with different architectures share and integrate information differently, specific structures or topologies of social networks synchronize or integrate knowledge in different ways. Future studies can combine experimental and computational approaches to study the coevolution of neural networks capable of processing large social network topologies, and the emergence of topologies that serve different collective functions and outcomes.

Combining experimental and computational approaches empowers researchers to investigate how the topology of social networks shapes collective cognition and behaviour and help design goal-directed social network topologies toward desired outcomes (e.g. correcting misinformation about a global pandemic or coordinating rapid vaccine discovery). Comparative studies on social and non-social graph learning across species could offer insight into the evolution of neural and social mechanisms in humans, other species and machines. Such an interdisciplinary approach empowers researchers to design effective multi-agent machine intelligence inspired by knowledge of human collective cognition. In turn, multi-agent machine learning models of collective intelligence can help theorize and test hypotheses about the coevolution of complex neural architectures and complex social networks.

## Data accessibility

This article has no additional data.
